# Distinct immune profiles of HIV‐infected subjects are linked to specific lipid mediator signature

**DOI:** 10.1002/iid3.629

**Published:** 2022-05-27

**Authors:** Magdalena Sips, Sarah Gerlo, Laura De Clercq, Esteban A. Gomez, Romain A. Colas, Jesmond Dalli, Linos Vandekerckhove

**Affiliations:** ^1^ Department of Internal Medicine and Pediatrics Ghent University Ghent Belgium; ^2^ Department of Biomolecular Medicine Ghent University Ghent Belgium; ^3^ Lipid Mediator Unit, Centre for Biochemical Pharmacology, William Harvey Research Institute, Barts and The London School of Medicine and Dentistry Queen Mary University of London London UK; ^4^ Centre for Inflammation and Therapeutic Innovation Queen Mary University of London London UK

**Keywords:** human, infections, inflammation, lipid mediators, viral/retroviral

## Abstract

**Introduction:**

To date, with no prophylactic human immunodeficiency virus (HIV) vaccine available, HIV incidence rates remain undefeated. Despite full virological suppression, HIV+ individuals exhibit a higher rate of cardiovascular disorders and cancers what is attributed to the residual, persistent levels of immune activation.

**Methods:**

We have established the Virological and Immunological Monitoring (VIM) platform and forty VIM samples that included treated immunological responders (IRs) or nonresponders (INRs), viremic untreated subjects and uninfected controls, were phenotyped by flow cytometry and plasma was used to quantify proinflammatory eicosanoids and the specialized proresolving mediators by liquid chromatography tandem mass spectrometry.

**Results:**

While HIV infection profoundly altered lipid mediator (LM) profile, differences were also seen in patients on viral suppressive therapy. IRs exhibited higher levels of proresolving mediators as compared to INRs and notable differences in plasma LM were also seen in early and late treated individuals.

**Conclusions:**

This study demonstrated distortions in proinflammatory/proresolution processes in infected patients including those with controlled viremia.

## Graphical abstract

Human immunodeficiency virus (HIV) infection profoundly altered lipid mediator profile in viremic patients and in patients on viral suppressive therapy. Higher CD4/CD8 ratio and early treatment were positively associated with proresolving levels of specialized proresolving mediators (SPMs), directly linking the presence of circulating SPMs to (near) normalized immune signature. Thus, HIV‐infected patients may benefit a therapeutic approach that aims to restore resolution pathways.

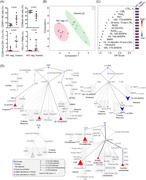

## 1 INTRODUCTION

Resolution of inflammation following the termination of the acute phase of infection is a main discriminator between nonpathogenic and pathogenic simian/human immunodeficiency virus (SIV/HIV) infection. Natural SIV hosts rapidly downmodulate inflammation at the end of acute infection and exhibit only transient robust proinflammatory signaling.^1^ HIV infection, however, leads to sustained unresolved inflammation and long‐lasting disrupted immune homeostasis is pronounced at the level of the innate and adaptive immune system.^2^

For many years resolution of inflammation was considered a passive process, where proinflammatory mediators dilute over time. The identification of essential fatty acid‐ derived proresolving mediators, termed specialized proresolving mediators (SPMs), in recent years, demonstrated that resolution of inflammation is an active process orchestrated via the tight regulation of cellular and biochemical processes, resulting in the restoration of immune homeostasis.^3^ SPMs consist of families of autacoids that include lipoxins, resolvins, protectins, and maresins. These autacoids are produced via the enzymatic conversion of essential fatty acids. SPMs actively reprogram the immune response to promote clearance of invading pathogens, and counter‐regulate the production of inflammation‐initiating molecules. Deficiency in resolution pathways is linked with multiple inflammatory disorders, including autoimmunity and infections.^4^, ^5^

We hypothesized that chronic HIV‐infection may be linked with a disruption of resolution pathways thereby contributing to dysregulated immune homeostasis. For this purpose, we measured peripheral blood proinflammatory and proresolving lipid mediators (LMs) in treatment‐naïve and antiretroviral therapy (ART)‐treated HIV‐infected patients and uninfected controls. To link LMs with cellular markers of immune activation, we phenotyped neutrophils, natural killer (NK) cells and cytotoxic T cells (CTLs) from study subjects. LM levels were then compared with clinical and viro‐immunological patient profiles to obtain insights into the possible associations between the proinflammatory/proresolution pathways and disease characteristics.

## 2 MATERIALS AND METHODS

### 2.1 Study subjects

The Ethical Committee of the University Hospital Ghent approved the study and each subject gave written informed consent. Study subjects included: 24 chronically infected HIV+ subjects on ART with good or poor immune reconstitution, that is, 17 immunological responders (IR), with optimal CD4 recovery >450 cells per mm^3^ of blood after at least 2 years of suppressive ART or 7 immunological nonresponders (INRs), with suboptimal CD4 + T‐cell recovery <400 cells per mm^3^ after at least 2 years of suppressive ART; 7 not yet treated viremic HIV+ subjects (VIREMIC) and 9 uninfected controls (HIV−). The IR group consisted of 8 subjects treated early in acute infection (IR early) or 9 subjects treated late in the chronic stage of infection (IR late). All but three ART‐treated individuals had undetectable viral load (VL). The viral blips in the three individuals were 92, 231, and 431 copies/ml. Study participants characteristics are depicted in Table S1.

### 2.2 Phenotypic profiling by multiparameter flow cytometry

Phenotypic profiling was performed on freshly isolated polymorphonuclear neutrophils (PMNs) and peripheral blood mononuclear cells (PBMCs) using multiparametric flow cytometry. PMNs (10^6^ per panel), isolated with a human neutrophil enrichment kit (Stem Cell), were stained for CD66b (clone G10F5), CD16 (clone 3G8), CD64 (clone 10.1), CD62L (DREG‐56), PD‐L1 (clone 29E.2A3), and HLA‐DR (clone L243). PBMCs (10^6^ per panel), isolated using Ficoll‐Hypaque density gradient centrifugation, were stained for CD4 (clone OKT4), CD8 (clone RPA‐T8), CD56 (clone NCAM16.2), CD16 (clone 3G8), CTLA4 (clone BIN3), Tim3 (clone 7D3), CD38 (clone HIT2), LAG3 (clone 3DS223H), PD1 (clone EH12.1), and HLA‐DR (clone L243). Data were acquired on a BD LSRII flow cytometer and analyzed using FlowJo software v10.

### 2.3 LM profiling

1 mL of freshly isolated plasma was placed in ice‐cold methanol and 500 pg of deuterium labeled internal standards were added the samples to facilitate identification and quantification. All samples were profiled using liquid chromatography tandem mass spectrometry (LC‐MS/MS) as previously described.^6^

### 2.4 Pathway analyses

Differences between concentrations of LMs from the different groups were expressed as the log_2_ fold change. Based on these differences, LM biosynthesis pathways were built using Cytoscape v3.7.1. Distinct LM families are highlighted according to the essential fatty substrate they originate from using distinct colors and their biosynthetic pathways using different line types. Up or down regulated mediators were denoted with using upward and downward facing triangles, respectively, and node size was used to depict the extent of differential expression.

### 2.5 Statistics

Nonparametric statistics were used for comparisons of immunological parameters. Reported *p* values are two sided and values <.05 were considered significant. To assess differences in LM profiles we used partial least squares‐discrimination analysis (PLS‐DA)^7^ that was performed using MetaboAnalyst^8^ and results were autoscaled. PLS‐DA is based on a linear multivariate model that identifies variables that contribute to class separation of observations on the basis of their variables (LM levels). During classification, observations were projected onto their respective class model. The score plot illustrates the systematic clusters among the observations (closer plots presenting higher similarity in the data matrix).

## 3 RESULTS

### 3.1 Distinct plasma LM profiles in HIV infected and noninfected volunteers

**Figure 1:**
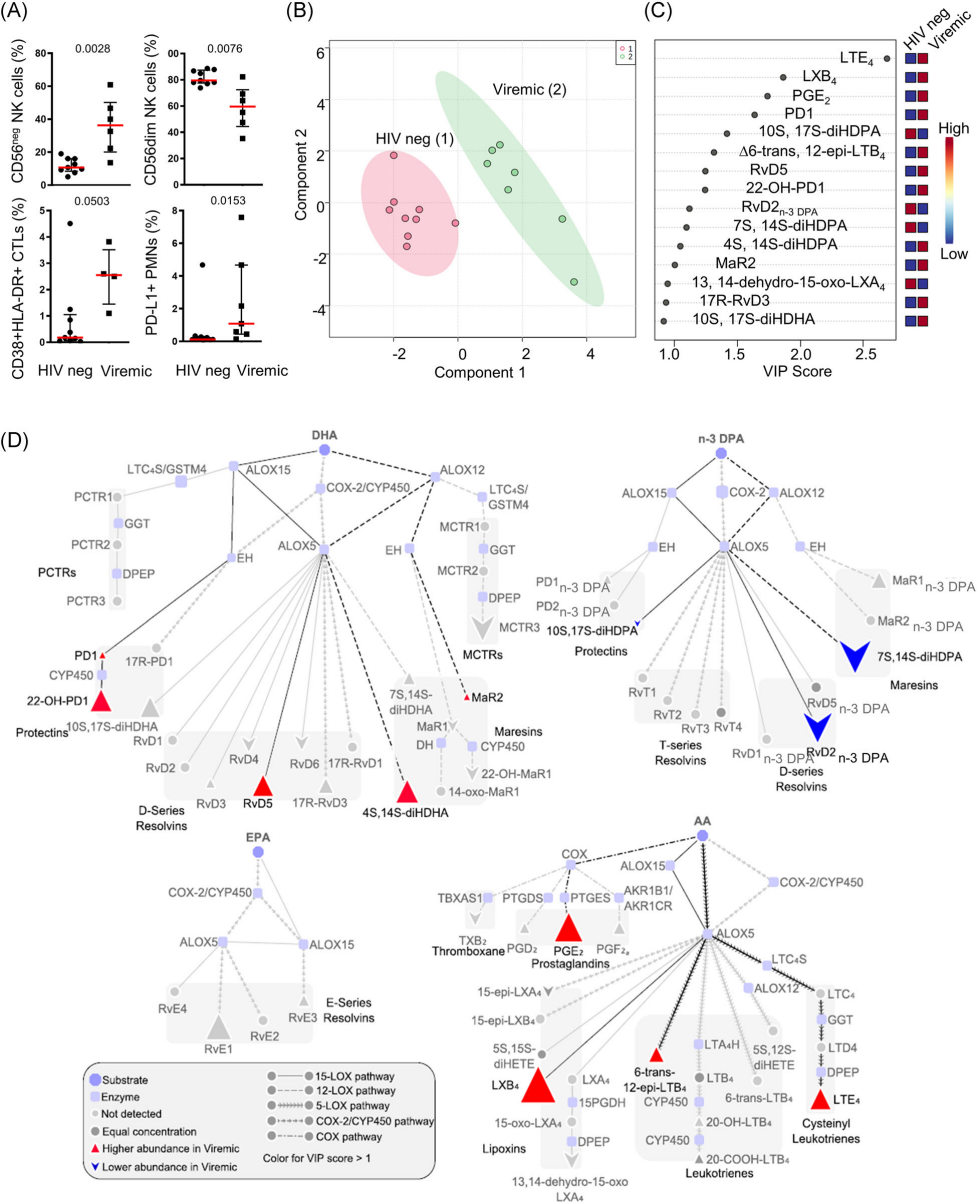
Immune signature and lipid mediator profile of HIV− and HIV+ subjects. White blood cells immunotyping revealed NK cell subset redistribution, activation of CTLs and PMNs in HIV+ viremicsubjects (A). PLS‐DA score plot of LM concentrations for HIV‐ and HIV+ viremic groups is illustrated in (B). Shaded regions in the score plot denote 95% confidence regions. Variable importance in projection (VIP) scores of 15 LMs with the greatest differences in concentrations between groups are shown in (C) and pathway analyses for the differential expression of mediators from the DHA, n‐3 DPA, EPA, and AA metabolomes are represented in (D). Results are from 7 HIV+ viremic patients and 9 HIV− patients. AA, arachidonic acid; CTL, cytotoxic T cell; CTL, cytotoxic T lymphocyte; DHA, docosahexaenoic acid; DPA, docosapentaenoic acid; HIV, human immunodeficiency virus; LM, lipid mediator; NK, natural killer; PMN, polymorphonuclear neutrophil

NK cell subset redistribution and an increase in frequency of CD38 + HLA‐DR + CTLs and PD‐L1 + suppressive PMNs were observed in the viremic group (Figure 1A), in accord with published findings.^9^, ^10^, ^11^ To evaluate the contribution of LM to the observed immunological changes we next evaluated plasma LMs concentrations measuring LM concentrations from the arachidonic acid (AA), n‐3 docosapentaenoic acid (n‐3 DPA), docosahexaenoic acid (DHA), and eicosapentaenoic acid (EPA) metabolomes. In plasma from both healthy volunteers and HIV+ patients we identified mediators from the cyclooxygenase and lipoxygenase pathways (Table S2). We next used PLS‐DA, a multivariate analysis that creates a linear regression model and accounts for multicollinearity to identify the relationship between samples, to evaluate whether plasma LM concentrations were different between these two groups. This analysis demonstrated that plasma LM concentrations in healthy volunteers were distinct from those measured in HIV+ viremic as demonstrated by a separation in the clusters representing each of these groups (Figure 1B). This shift was linked with a differential regulation of both proresolving mediators, including LXB_4_, PD1, and RvD5, as well as inflammatory eicosanoids such as LTE_4_ and PGE_2_ in plasma from viremic patients (Figure 1C) all of which display variable importance in projection (VIP) scores >1. These scores identify the mediators that contribute mostly to the observed separation between the two groups. To obtain further insights into the impact of HIV infection on the regulation of peripheral blood LMs and to evaluate whether specific biosynthetic pathways and/or enzymes display altered activities in HIV+ viremic patients, we conducted an LM pathway analysis focusing on the mediators that displayed greatest differences between the two groups as denoted by a VIP score >1 in the multivariate analysis. This uncovered an increase in ALOX5 and ALOX15‐derived products from DHA and AA metabolomes in viremic patients that included the proresolving mediators PD1 and its bioactive further metabolite 22‐OH‐PD1 as well as the AA‐derived eicosanoid LTE_4_ (Figure 1D). Intriguingly this analysis also revealed a downregulation of the n‐3 DPA derived products of these enzymes including the pro‐resolving mediator RvD2_n‐3 DPA_. COX activity was also increased in viremic patients, as illustrated by an upregulation of the immune‐suppressive mediator PGE_2_ (Figure 1D).

### 3.2 Downregulation of specific SPMs including antiviral PD1 in plasma of INRs

**Figure 2:**
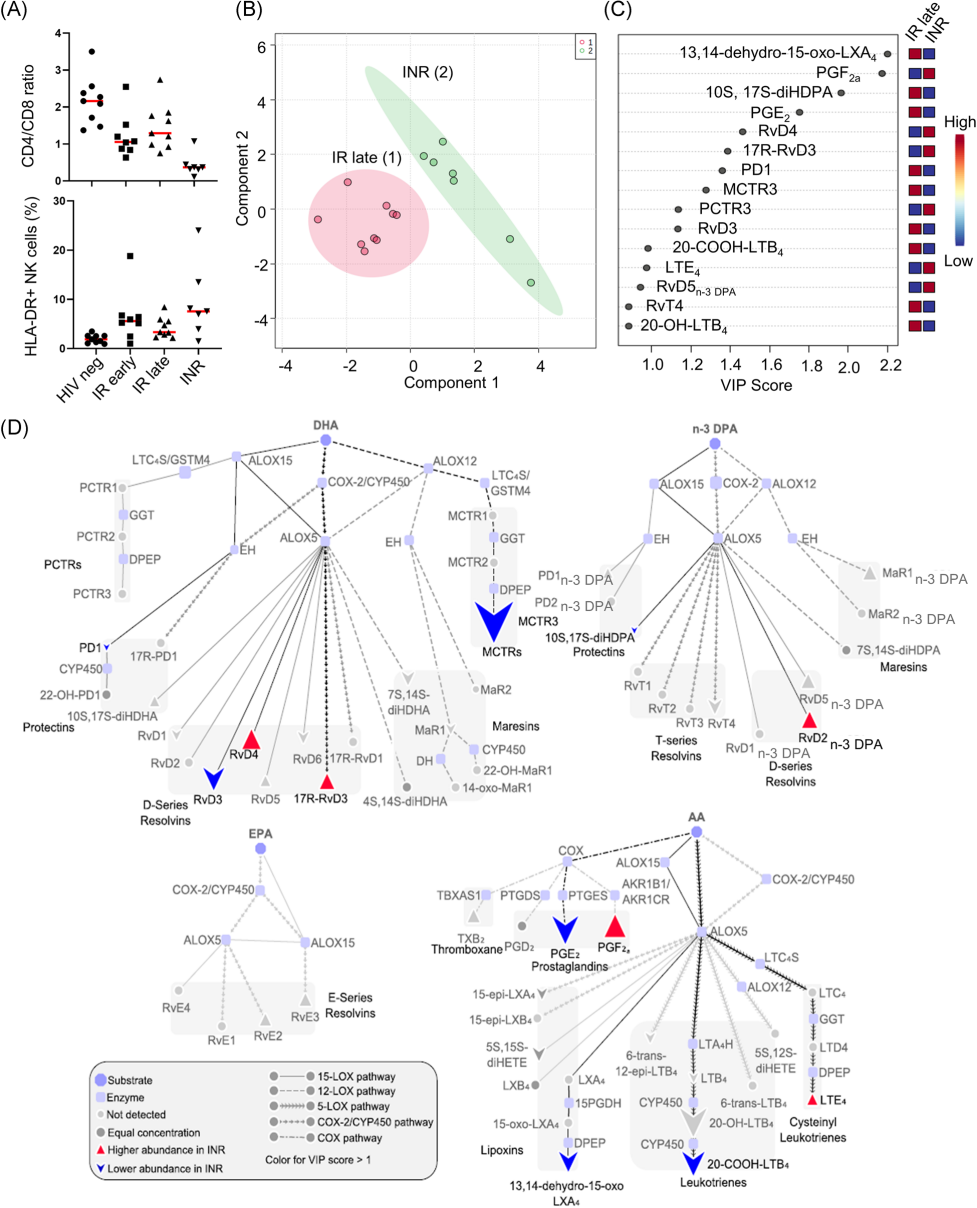
Immune signature and lipid mediator profile in immunological responders (IR) and immunological nonresponders (INR). CD4/CD8 ratio and frequency of activated HLA‐DR + NK cells is depicted in (A). PLS‐DA score plot of LM concentrations for INR and IR late groups is illustrated in (B). Shaded regions in the score plot denote 95% confidence regions. Variable importance in projection (VIP) scores of 15 LMs with the greatest differences in concentrations between groups are shown in (C) and pathway analyses for the differential expression of mediators from the DHA, n‐3 DPA, EPA, and AA metabolomes are represented in (D). Results are from 7 INR and 9 IR late patients. AA, arachidonic acid; EPA, eicosapentaenoic acid; INR, immunological nonresponder; IR, immunological responder; LM, lipid mediator; NK, natural killer

Having observed alterations in LM pathways in HIV‐infected viremic individuals, we next sought to address whether the LM concentrations change following ART therapy. Upon ART‐therapy, most HIV+ patients recover CD4 + T cells, however, about 25% of patients, often late presenters, fail to do so. These subjects are among the highest risk group for developing comorbidities as a low CD4/CD8 ratio is an immune risk phenotype and is linked to immune senescence not only in HIV‐infected subjects.^13^ Because INRs display low CD4 count and often an activated NK cell compartment^14^ (Figure 2A), we were poised to investigate whether there was a link between immunological recovery and the regulation of peripheral LMs. Multivariate analysis demonstrated that plasma LM profiles of INRs clustered separately from those obtained from late‐treated responder (IR late; Figure 2B). This was linked with a differential regulation of 10 mediators that had VIP scores greater than 1 and included the proresolving mediators including PD1 which displays potent antiviral activities^15^ (Figure 2C and Table S3). Pathway analysis of mediators differentially regulated between INR and IR late highlighted an upregulation of ALOX5‐ALOX15 interaction products from the DHA and n‐3 DPA metabolomes in INRs as confirmed by an upregulation in the concentrations of several D‐series resolvins including RvD4 from the DHA metabolome and RvD2_n‐3 DPA_ from the n‐3 DPA metabolome (Figure 2D). Notably, we observed a downregulation of AA‐derived ALOX5‐ALOX15 interaction produces from the AA‐metabolome as demonstrated by a decrease in 13,14‐dehydro‐15‐oxo‐LXA_4_, the further metabolite of LXA_4_ (Figure 2D). These changes were coupled with an apparent shunting of ALOX5 activity away from LTB_4_ formation towards the production of LTE_4_ in these patients, suggesting a shift in the coupling of ALOX5 from LTA_4_ hydrolase to LTC_4_ synthase. A similar shift in activity was observed when assessing PG concentrations where we observed a downregulation of PGE_2_ and a concomitant increase in PGF_2a_ concentrations in INR, suggesting a selective coupling of COX enzymes with synthases involved in the formation of PGF_2a_. Thus, next to disruption of lymphatic tissue architecture^16^ as well as an autoimmune‐like responses,^17^ both documented in INRs, our results identify another layer of immune mediators present in pathology of these subjects despite undetectable VL.

### 3.3 Treatment of HIV+ patients during the acute phase upregulates a distinct group of SPMs

**Figure 3:**
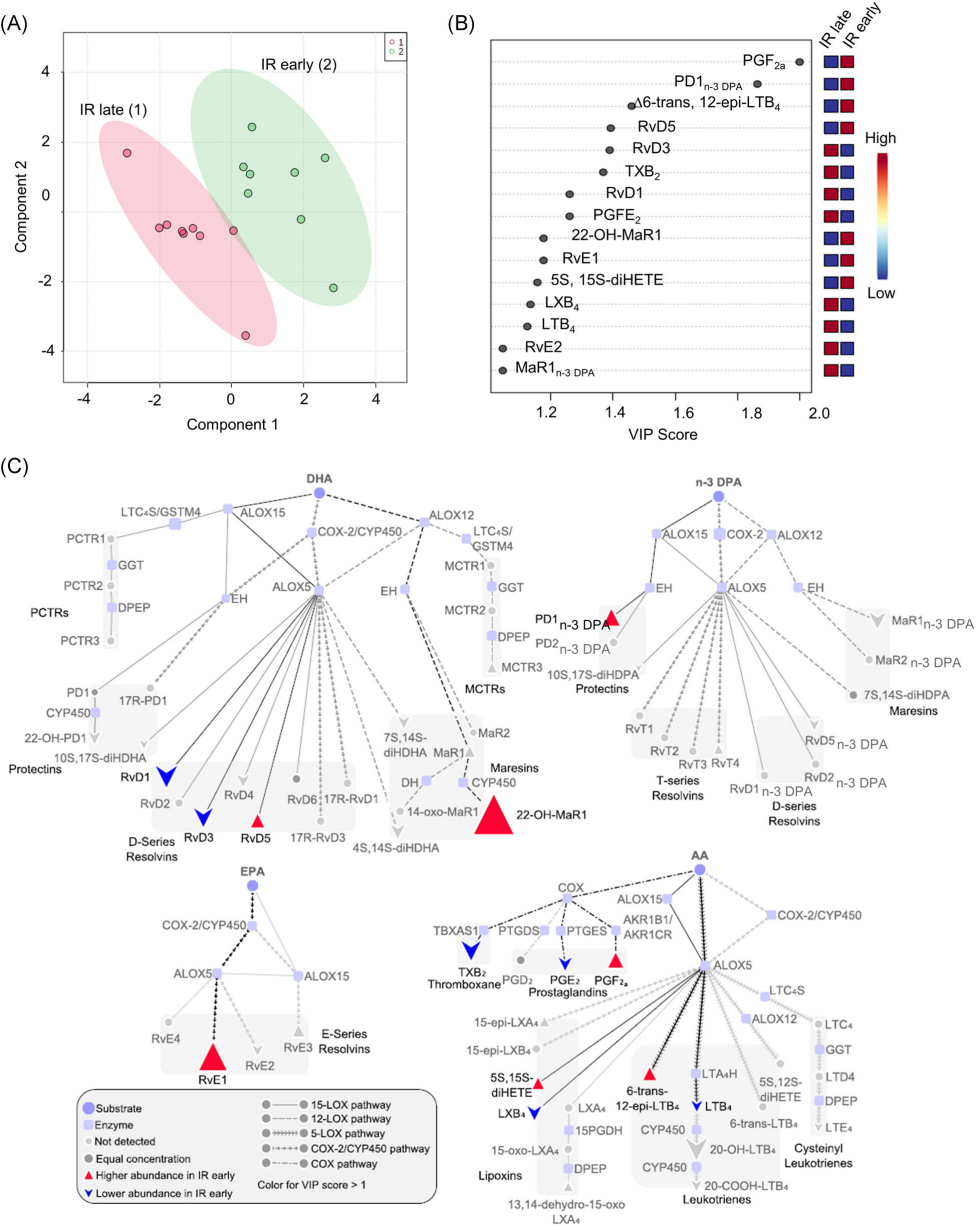
Lipid mediator profile in immunological responders treated in acute (IR early) or chronic (IR late) stage of infection. PLS‐DA score plot of LM concentrations for IR early and IR late groups is illustrated in (A). Shaded regions in the score plot denote 95% confidence regions. Variable importance in projection (VIP) scores of 15 LMs with the greatest differences in concentrations between groups are shown in (B) and pathway analyses for the differential expression of mediators from the DHA, n‐3 DPA, EPA, and AA metabolomes are represented in (C). Results are from 8 IR early patients and 9 IR late. AA, arachidonic acid; DHA, docosahexaenoic acid; DPA, docosapentaenoic acid; EPA, eicosapentaenoic acid; IR, immunological responder; LM, lipid mediator; PLS‐DA, partial least squares‐discrimination analysis

Current guidelines on initiation of ART promote the treatment of all HIV+ individuals regardless of CD4 count. While there is mounting evidence of the effect of early ART on the course of infection, the clinical and immunological benefits of this approach remain to be defined. Assessment of LM profiles demonstrated that plasma SPMs in patients treated during the acute phase (IR early) were markedly distinct from those treated in the chronic phase (IR late) (Figure 3A). Assessment of VIP scores demonstrated that more than 13 LMs were differentially expressed between the two treatment groups with an upregulation of mediators from all three omega‐3 fatty acid metabolomes including the DHA‐derived RvD5, and the n‐3 DPA‐derived PD1_n‐3 DPA_ (Figure 3B). Pathway analysis suggested a selective regulation of ALOX and COX activity in IR early patients compared to IR late patients. Indeed, we observed an increase in PGF_2a_ with a concomitant decrease in both COX‐derived PGE_2_ and TXB_2_, suggesting a selective shunting of the common biosynthetic intermediate PGH_2_ towards the former mediator. A similar pattern was observed for the ALOX5‐ALOX15 interaction products from the AA and DHA metabolomes. Here we observed an increase in RvD5 and 5S, 15S‐di‐HETE concentrations and a decrease in the concentrations of RvD1, RvD3, and LXB_4_, suggesting that the biosynthetic intermediates within each of the pathways are being preferentially converted to the double lipoxygenation products in IR early patients. This pathway analysis also highlighted an increased activity of ALOX15 in the n‐3 DPA pathway in the IR early group, as demonstrated by an upregulation of PD1_n‐3 DPA_ concentrations as well as an increase in the CYP450‐ALOX5 interaction product RvE1 (Figure 3C). Interestingly, both RvE1 and protectins are implicated in the regulation of host responses to viral infections.^15^, ^18^

## 4 DISCUSSION

Our study for the first time comprehensively examined the circulating levels of LMs in HIV‐infected patients and uninfected controls and revealed a significant increase in the proinflammatory AA‐derived LTE_4_ as well as a decrease in several DHA and n‐3 DPA‐derived SPMs in viremic patients. Furthermore, CD4/CD8 ratio as well as time of treatment initiation were discriminators of LM profiles, such that higher CD4/CD8 ratio and early treatment were positively associated with proresolving levels of SPMs, directly linking the presence of circulating SPMs to (near) normalized immune signature. Thus, notwithstanding the direct effect of ART therapy on replicating virus, patients chronically infected with HIV, especially INRs, may benefit a therapeutic approach that aims to restore resolution pathways. This could be achieved via the use of analogues and/or mimetics to SPM that reprogram immune responses to interrupt the chronic inflammatory cycle and restore homeostasis.^19^ Another approach that has recently garnered interest is the use of immunonutrition. Up to date, several trials have attempted to test the hypothesis of a beneficial effect of fish oil supplements on immune parameters, however, with variable outcomes^20^, ^21^ likely owing to inadequate dosage and conditions of use. In this context we found that well‐designed administration of omega‐3 enriched therapeutics upregulates endogenous SPM production in both healthy volunteers and patients with chronic inflammatory diseases and reprograms the peripheral blood cell transcriptome.^22^, ^23^

Recently, severe SARS‐CoV‐2 infection has been linked to a unique milieu of LMs with the loss of immune‐regulatory PGs and RvE3 and the increased products of ALOX5 and CYP^24^ while another study highlighted rise of proinflammatory eicosanoids and docosanoids in the lungs of intubated COVID19 patients.^25^ Similarly, upregulation of the inflammatory eicosanoids and decrease in the production of a number of SPMs in severe tuberculosis infection highlights major distortions in LMs in potentially lethal infections hallmarked by dysregulated immune response.^26^ While there has been a steady progress in studying LMs in serious infections, identification of disrupted LM pathways requires more in depth understanding how molecular and cellular pathways interplay to regulate LM levels to devise new therapeutic opportunities to control severe infections or inflammatory disorders of autoimmune origin.^6^

Apart from the novel insights that the present study offers into the pathophysiology of HIV infections, there are also some limitations that need to be taken into consideration. These include the lack of a causal link between the changes in specific LMs and the observed changes in immune responses. Such studies will need to evaluate whether, for example all the SPM found to be upregulated in patients that are responsive to ART therapy are directly responsible for the observed improvement in immunological responses or whether this could be potentially via the activation of secondary mechanisms. Furthermore, given the limited sample size, in the present study we were unable to account for potential confounders, such as for example comorbidities and duration of infection before diagnosis. Future studies, in larger patient populations that take these and other potential confounders into account, will need to be conducted to establish the potential role for plasma SPM concentrations in both the pathology of HIV infections as well as in the responsiveness to ART therapy. Beyond cross‐sectional analysis, longitudinal probing of patients' peripheral blood starting in acute infection could provide critical insights into kinetic changes of LMs during the course of infection and response to ART.

Together the present findings shed new light on the potential contribution of LMs to pathophysiology of HIV infections. They also demonstrate that patients that are responsive to ART display distinct plasma LM concentrations suggesting that the regulation of these molecules may be linked to immunological responses. Thus, the present findings lend support to a potential utility of these molecules in HIV patient stratification.

## CONFLICT OF INTERESTS

JD, EAG and RAC are inventors on patents related to the composition of matter and/or use of pro‐resolving mediators some of which are licensed by Brigham and Women' Hospital or Queen Mary University of London for clinical development. JD is also scientific founder and director of Resolomics Ltd. The remaining authors declare that there are no conflict of interests.

## AUTHOR CONTRIBUTIONS

**Magdalena Sips Jesmond Dalli** and **Linos Vandekerckhove:** designed the experiments and conceived the overall research plan; **Magdalena Sips, Laura De Clercq, Esteban A. Gomez,** and **Romain A. Colas:** conducted the experiments and/or analyzed results. **Sarah Gerlo:** contributed to data interpretation and all authors contributed to manuscript preparation.

### DATA AVAILABILITY STATEMENT

The data that support the findings of this study are available from the corresponding author upon reasonable request.

## Supporting information

Supporting information.
