# Supplementary material for: Distinct immune profiles of HIV‐infected subjects are linked to specific lipid mediator signature
Source: Immun Inflamm Dis. 2022 May 27;10(6):e629. doi: 10.1002/iid3.629 (PMC9138705; doi:10.1002/iid3.629)
Supplement: Supplementary file 1 — Supporting information. [file IID3-10-e629-s001.docx]

**Supplemental Table 1.** Clinical characteristics of VIM study participants. Data are median (interquartile range).

| Characteristic | HIV NEG (Uninfected Controls) | INR (Immunological Non-Responders) | IR early (Immunological responders, early treated) | IR late (Immunological responders, late treated) | Viremic (Untreated patients) |
| --- | --- | --- | --- | --- | --- |
| Number of subjects | 9 | 7 | 8 | 9 | 7 |
| Gender | male only | male only | male only | male only | male only |
| Age, y | 39 (26-65) | 52 (48-59) | 34 (27-50) | 43 (26-57) | 44 (25-49) |
| CD4+ T-cell count, cells/µl | 711 (455-1060) | 246 (61-363) | 657 (453-1430) | 677 (474-1250) | 487 (29-972) |
| CD4+ T cell frequency [%] | 44.6 (41.40-49.10) | 16.7 (11.70-18.90) | 37.5 (32.63-45.68) | 41.2 (35.75-47.50) | 27.5 (26.90-31.00) |
| CD8+ cytotoxic T cell frequency [%] | 21.2 (19.20-25.35) | 42 (37.60-50.80) | 36.9 (30.95-39.95) | 31 (26.30-34.75) | 52 (45.20-58.90) |
| CD4/CD8 ratio | 2.16 (1.59-2.47) | 0.37 (0.31-0.44) | 1.05 (0.85-1.44) | 1.29 (0.95-1.8) | 0.61 (0.46-0.63) |

**Supplemental Table 2: Altered LM profiles in plasma from Viremic patients**

|  | **HIV Neg (pg/mL)** | | | **Viremic (pg/mL)** | | |
| --- | --- | --- | --- | --- | --- | --- |
| **DHA Bioactive Metabolome** | **Mean** | ± | **SEM** | **Mean** | ± | **SEM** |
| RvD1 |  | - |  |  | - |  |
| RvD2 |  | - |  |  | - |  |
| RvD3 |  | - |  |  | - |  |
| RvD4 | 3.06 | ± | 1.52 | 1.80 | ± | 1.27 |
| RvD5 | 0.69 | ± | 0.50 | 3.64 | ± | 2.44 |
| RvD6 | 2.36 | ± | 0.69 | 0.72 | ± | 0.78 |
| 17R-RvD1 | 1.73 | ± | 0.95 | 0.95 | ± | 1.02 |
| 17R-RvD3 | 4.15 | ± | 1.82 | 2.66 | ± | 1.97 |
|  |  |  |  |  |  |  |
| PD1 | 2.18 | ± | 0.84 | 5.07 | ± | 1.74 |
| 17R-PD1 |  | - |  |  | - |  |
| 10S,17SdiHDHA | 0.48 | ± | 0.35 | 1.64 | ± | 1.77 |
| 22-OH-PD1 | 1.34 | ± | 0.96 | 4.00 | ± | 2.80 |
|  |  |  |  |  |  |  |
| PCTR1 |  | - |  |  | - |  |
| PCTR2 |  | - |  |  | - |  |
| PCTR3 | 0.38 | ± | 0.40 | 0.57 | ± | 0.62 |
|  |  |  |  |  |  |  |
| MaR1 | 11.42 | ± | 4.41 | 11.37 | ± | 9.18 |
| MaR2 |  | - |  | 0.65 | ± | 0.71 |
| 22-OH-MaR1 | 30.90 | ± | 28.49 | 16.80 | ± | 18.15 |
| 14-oxo-MaR1 |  | - |  |  | - |  |
| 7S,14S diHDHA | 7.20 | ± | 1.84 | 4.58 | ± | 2.05 |
| 4S,14S-diHDHA | 0.55 | ± | 0.58 | 3.11 | ± | 2.40 |
|  |  |  |  |  |  |  |
| MCTR1 |  | - |  |  | - |  |
| MCTR2 |  | - |  |  | - |  |
| MCTR3 |  | - |  |  | - |  |
|  |  |  |  |  |  |  |
| **n-3 DPA Bioactive Metabolome** |  |  |  |  |  |  |
| RvT1 | 1.55 | ± | 1.10 | 2.70 | ± | 2.26 |
| RvT2 |  | - |  |  | - |  |
| RvT3 |  | - |  |  | - |  |
| RvT4 | 0.80 | ± | 0.57 | 0.40 | ± | 0.43 |
|  |  |  |  |  |  |  |
| RvD1_n3 DPA_ | 0.40 | ± | 0.43 |  | - |  |
| RvD2_n3 DPA_ |  | - |  | 1.79 | ± | 1.32 |
| RvD5_n3 DPA_ | 5.85 | ± | 6.20 | 5.57 | ± | 2.66 |
|  |  |  |  |  |  |  |
| PD1_n3 DPA_ | 1.01 | ± | 0.58 | 0.66 | ± | 0.71 |
| 10S,17S-diHDPA | 0.21 | ± | 0.22 |  | - |  |
|  |  |  |  |  |  |  |
| MaR1_n3 DPA_ | 0.66 | ± | 0.70 | 1.80 | ± | 1.26 |
| 7S,14S-diHDPA | 68.20 | ± | 46.21 | 14.27 | ± | 11.17 |
|  |  |  |  |  |  |  |
| **EPA Bioactive Metabolome** |  |  |  |  |  |  |
| RvE1 | 1.90 | ± | 2.02 | 12.51 | ± | 13.51 |
| RvE2 |  | - |  |  | - |  |
| RvE3 | 12.32 | ± | 4.50 | 13.95 | ± | 8.68 |
|  |  |  |  |  |  |  |
| **AA Bioactive Metabolome** |  |  |  |  |  |  |
| LXA_4_ | 0.53 | ± | 0.34 | 0.20 | ± | 0.21 |
| LXB_4_ |  | - |  | 2.37 | ± | 1.65 |
| 5,15-diHETE | 46.29 | ± | 11.06 | 45.65 | ± | 12.62 |
| 15-epi-LXA_4_ |  | - |  | 0.55 | ± | 0.59 |
| 15-epi-LXB_4_ | 0.47 | ± | 0.50 | 3.57 | ± | 3.86 |
| 13,14-dehydro-15-oxo-LXA_4_ | 1.44 | ± | 0.58 | 0.67 | ± | 0.72 |
| 15-oxo-LXA_4_ | 0.09 | ± | 0.10 |  | - |  |
|  |  |  |  |  |  |  |
| LTB_4_ | 3.39 | ± | 0.63 | 3.16 | ± | 1.28 |
| 5,12 diHETE | 0.77 | ± | 0.27 | 0.85 | ± | 0.59 |
| 6-trans-LTB_4_ | 0.13 | ± | 0.14 |  | - |  |
| 12-epi-6-trans-LTB_4_ | 0.62 | ± | 0.26 | 0.71 | ± | 0.40 |
| 20-OH-LTB_4_ |  | - |  | 2.10 | ± | 2.27 |
| 20-COOH-LTB_4_ | 2.03 | ± | 1.55 | 1.91 | ± | 1.34 |
|  |  |  |  |  |  |  |
| LTC_4_ |  | - |  |  | - |  |
| LTD_4_ |  | - |  |  | - |  |
| LTE_4_ | 0.83 | ± | 0.38 | 3.54 | ± | 0.74 |
|  |  |  |  |  |  |  |
| PGD_2_ | 5.11 | ± | 1.47 | 1.10 | ± | 1.18 |
| PGE_2_ | 0.73 | ± | 0.78 | 1.53 | ± | 1.07 |
| PGF_2a_ | 9.22 | ± | 4.53 | 13.50 | ± | 5.47 |
| TXB_2_ | 3.51 | ± | 1.50 | 4.81 | ± | 1.87 |

Lipid mediators were extracted, identified and quantified in plasma from HIV negative and Viremic patients using Lipid Mediator profiling. Results are mean ± SEM. n = 9 HIV neg and 7 Viremic patients. - = Below limits of the assay

**Supplemental Table 3: Plasma lipid mediator concentrations in IR Late and INRs.**

|  | **IR Late (pg/mL)** | | | **INR (pg/mL)** | | |
| --- | --- | --- | --- | --- | --- | --- |
| **DHA Bioactive Metabolome** | **Mean** | ± | **SEM** | **Mean** | ± | **SEM** |
| RvD1 | 3.26 | ± | 1.94 |  | - |  |
| RvD2 | 0.66 | ± | 0.70 |  | - |  |
| RvD3 |  | - |  |  | - |  |
| RvD4 | 1.92 | ± | 1.03 | 0.48 | ± | 0.51 |
| RvD5 | 1.12 | ± | 0.65 | 2.65 | ± | 1.17 |
| RvD6 | 1.04 | ± | 0.58 | 1.49 | ± | 1.05 |
| 17R-RvD1 | 0.89 | ± | 0.71 | 0.81 | ± | 0.87 |
| 17R-RvD3 | 1.01 | ± | 0.53 | 0.56 | ± | 0.39 |
|  |  |  |  |  |  |  |
| PD1 | 2.35 | ± | 0.93 | 3.16 | ± | 1.08 |
| 17R-PD1 |  | - |  |  | - |  |
| 10S,17SdiHDHA | 0.35 | ± | 0.26 | 0.23 | ± | 0.25 |
| 22-OH-PD1 | 0.62 | ± | 0.50 |  | - |  |
|  |  |  |  |  |  |  |
| PCTR1 |  | - |  |  | - |  |
| PCTR2 |  | - |  |  | - |  |
| PCTR3 | 0.93 | ± | 0.69 |  | - |  |
|  |  |  |  |  |  |  |
| MaR1 | 10.90 | ± | 3.62 | 1.51 | ± | 1.61 |
| MaR2 |  | - |  |  | - |  |
| 22-OH-MaR1 |  | - |  | 14.40 | ± | 15.39 |
| 14-oxo-MaR1 |  | - |  |  | - |  |
| 7S,14S diHDHA | 6.08 | ± | 1.51 | 6.03 | ± | 2.93 |
| 4S,14S-diHDHA | 0.59 | ± | 0.62 |  | - |  |
|  |  |  |  |  |  |  |
| MCTR1 |  | - |  |  | - |  |
| MCTR2 |  | - |  |  | - |  |
| MCTR3 | 1.08 | ± | 1.13 | 1.32 | ± | 1.41 |
|  |  |  |  |  |  |  |
| **n-3 DPA Bioactive Metabolome** |  |  |  |  |  |  |
| RvT1 | 0.49 | ± | 0.52 | 1.15 | ± | 1.23 |
| RvT2 |  | - |  | 0.45 | ± | 0.48 |
| RvT3 | 0.31 | ± | 0.22 |  | - |  |
| RvT4 | 0.35 | ± | 0.37 | 0.57 | ± | 0.61 |
|  |  |  |  |  |  |  |
| RvD1_n3 DPA_ |  | - |  |  | - |  |
| RvD2_n3 DPA_ | 4.34 | ± | 1.54 | 0.85 | ± | 0.91 |
| RvD5_n3 DPA_ | 3.29 | ± | 1.79 | 2.51 | ± | 1.78 |
|  |  |  |  |  |  |  |
| PD1_n3 DPA_ | 0.15 | ± | 0.16 | 2.15 | ± | 1.04 |
| 10S,17S-diHDPA | 1.12 | ± | 0.49 | 0.45 | ± | 0.49 |
|  |  |  |  |  |  |  |
| MaR1_n3 DPA_ | 2.13 | ± | 1.61 |  | - |  |
| 7S,14S-diHDPA | 13.10 | ± | 3.86 | 15.33 | ± | 4.80 |
|  |  |  |  |  |  |  |
| **EPA Bioactive Metabolome** |  |  |  |  |  |  |
| RvE1 |  | - |  | 8.42 | ± | 6.84 |
| RvE2 | 0.33 | ± | 0.35 |  | - |  |
| RvE3 | 5.50 | ± | 3.70 | 8.59 | ± | 3.59 |
|  |  |  |  |  |  |  |
| **AA Bioactive Metabolome** |  |  |  |  |  |  |
| LXA_4_ | 0.75 | ± | 0.54 |  | - |  |
| LXB_4_ | 3.83 | ± | 1.75 | 3.32 | ± | 2.46 |
| 5,15-diHETE | 41.28 | ± | 7.65 | 55.25 | ± | 20.01 |
| 15-epi-LXA_4_ | 0.71 | ± | 0.44 | 0.85 | ± | 0.68 |
| 15-epi-LXB_4_ |  | - |  |  | - |  |
| 13,14-dehydro-15-oxo-LXA_4_ | 0.74 | ± | 0.36 | 1.34 | ± | 0.94 |
| 15-oxo-LXA_4_ |  | - |  | 0.19 | ± | 0.20 |
|  |  |  |  |  |  |  |
| LTB_4_ | 2.29 | ± | 0.51 | 1.74 | ± | 0.72 |
| 5,12 diHETE | 0.14 | ± | 0.15 | 0.16 | ± | 0.17 |
| 6-trans-LTB_4_ |  | - |  |  | - |  |
| 12-epi-6-trans-LTB_4_ | 0.75 | ± | 0.35 | 0.35 | ± | 0.26 |
| 20-OH-LTB_4_ |  | - |  |  | - |  |
| 20-COOH-LTB_4_ | 0.98 | ± | 0.78 | 1.19 | ± | 0.85 |
|  |  |  |  |  |  |  |
| LTC_4_ |  | - |  |  | - |  |
| LTD_4_ |  | - |  |  | - |  |
| LTE_4_ | 1.38 | ± | 0.39 | 1.13 | ± | 0.51 |
|  |  |  |  |  |  |  |
| PGD_2_ | 2.56 | ± | 0.76 | 2.73 | ± | 1.69 |
| PGE_2_ | 2.77 | ± | 1.01 | 2.42 | ± | 1.04 |
| PGF_2a_ | 6.55 | ± | 2.11 | 13.27 | ± | 3.94 |
| TXB_2_ | 6.95 | ± | 4.08 | 1.01 | ± | 0.72 |

Lipid mediators were extracted, identified and quantified in plasma from IR Late and INR patients using Lipid Mediator profiling. Results are mean ± SEM. n = 9 IR Late and 7 INR patients. - = Below limits of the assay

**Supplemental Table 4: Plasma lipid mediator concentrations in IR Late and IR early.**

|  | **IR late (pg/mL)** | | | **IR early (pg/mL)** | | |
| --- | --- | --- | --- | --- | --- | --- |
| **DHA Bioactive Metabolome** | **Mean** | ± | **SEM** | **Mean** | ± | **SEM** |
| RvD1 | 3.26 | ± | 1.94 | 1.72 | ± | 1.33 |
| RvD2 | 0.66 | ± | 0.70 |  | - |  |
| RvD3 |  | - |  |  | - |  |
| RvD4 | 1.92 | ± | 1.03 | 5.24 | ± | 3.28 |
| RvD5 | 1.12 | ± | 0.65 | 1.30 | ± | 0.72 |
| RvD6 | 1.04 | ± | 0.58 | 1.08 | ± | 0.78 |
| 17R-RvD1 | 0.89 | ± | 0.71 | 0.39 | ± | 0.42 |
| 17R-RvD3 | 1.01 | ± | 0.53 | 0.36 | ± | 0.39 |
|  |  |  |  |  |  |  |
| PD1 | 2.35 | ± | 0.93 | 1.93 | ± | 0.79 |
| 17R-PD1 |  | - |  |  | - |  |
| 10S,17SdiHDHA | 0.35 | ± | 0.26 | 0.28 | ± | 0.30 |
| 22-OH-PD1 | 0.62 | ± | 0.50 | 0.55 | ± | 0.59 |
|  |  |  |  |  |  |  |
| PCTR1 |  | - |  |  | - |  |
| PCTR2 |  | - |  |  | - |  |
| PCTR3 | 0.93 | ± | 0.69 |  | - |  |
|  |  |  |  |  |  |  |
| MaR1 | 10.90 | ± | 3.62 | 7.45 | ± | 6.34 |
| MaR2 |  | - |  |  | - |  |
| 22-OH-MaR1 |  | - |  |  | - |  |
| 14-oxo-MaR1 |  | - |  |  | - |  |
| 7S,14S diHDHA | 6.08 | ± | 1.51 | 5.64 | ± | 1.01 |
| 4S,14S-diHDHA | 0.59 | ± | 0.62 | 0.54 | ± | 0.59 |
|  |  |  |  |  |  |  |
| MCTR1 |  | - |  |  | - |  |
| MCTR2 |  | - |  |  | - |  |
| MCTR3 | 1.08 | ± | 1.13 |  | - |  |
|  |  |  |  |  |  |  |
| **n-3 DPA Bioactive Metabolome** |  |  |  |  |  |  |
| RvT1 | 0.49 | ± | 0.52 | 3.05 | ± | 2.54 |
| RvT2 |  | - |  |  | - |  |
| RvT3 | 0.31 | ± | 0.22 | 0.34 | ± | 0.36 |
| RvT4 | 0.35 | ± | 0.37 |  | - |  |
|  |  |  |  |  |  |  |
| RvD1_n3 DPA_ |  | - |  |  | - |  |
| RvD2_n3 DPA_ | 4.34 | ± | 1.54 | 0.93 | ± | 1.01 |
| RvD5_n3 DPA_ | 3.29 | ± | 1.79 | 6.62 | ± | 3.27 |
|  |  |  |  |  |  |  |
| PD1_n3 DPA_ | 0.15 | ± | 0.16 | 0.53 | ± | 0.37 |
| 10S,17S-diHDPA | 1.12 | ± | 0.49 | 0.91 | ± | 0.48 |
|  |  |  |  |  |  |  |
| MaR1_n3 DPA_ | 2.13 | ± | 1.61 | 1.87 | ± | 1.01 |
| 7S,14S-diHDPA | 13.10 | ± | 3.86 | 19.74 | ± | 8.22 |
|  |  |  |  |  |  |  |
| **EPA Bioactive Metabolome** |  |  |  |  |  |  |
| RvE1 |  | - |  |  | - |  |
| RvE2 | 0.33 | ± | 0.35 | 0.56 | ± | 0.60 |
| RvE3 | 5.50 | ± | 3.70 | 9.63 | ± | 7.63 |
|  |  |  |  |  |  |  |
| **AA Bioactive Metabolome** |  |  |  |  |  |  |
| LXA_4_ | 0.75 | ± | 0.54 | 0.53 | ± | 0.46 |
| LXB_4_ | 3.83 | ± | 1.75 | 4.73 | ± | 1.94 |
| 5,15-diHETE | 41.28 | ± | 7.65 | 25.66 | ± | 7.79 |
| 15-epi-LXA_4_ | 0.71 | ± | 0.44 | 0.59 | ± | 0.37 |
| 15-epi-LXB_4_ |  | - |  | 0.91 | ± | 0.99 |
| 13,14-dehydro-15-oxo-LXA_4_ | 0.74 | ± | 0.36 | 0.12 | ± | 0.12 |
| 15-oxo-LXA_4_ |  | - |  | 1.19 | ± | 0.49 |
|  |  |  |  |  |  |  |
| LTB_4_ | 2.29 | ± | 0.51 | 2.21 | ± | 0.61 |
| 5,12 diHETE | 0.14 | ± | 0.15 |  | - |  |
| 6-trans-LTB_4_ |  | - |  |  | - |  |
| 12-epi-6-trans-LTB_4_ | 0.75 | ± | 0.35 | 0.56 | ± | 0.23 |
| 20-OH-LTB_4_ |  | - |  |  | - |  |
| 20-COOH-LTB_4_ | 0.98 | ± | 0.78 | 0.22 | ± | 0.24 |
|  |  |  |  |  |  |  |
| LTC_4_ |  | - |  |  | - |  |
| LTD_4_ |  | - |  |  | - |  |
| LTE_4_ | 1.38 | ± | 0.39 | 2.08 | ± | 0.68 |
|  |  |  |  |  |  |  |
| PGD_2_ | 2.56 | ± | 0.76 | 4.38 | ± | 1.60 |
| PGE_2_ | 2.77 | ± | 1.01 | 1.52 | ± | 0.94 |
| PGF_2a_ | 6.55 | ± | 2.11 | 30.36 | ± | 11.73 |
| TXB_2_ | 6.95 | ± | 4.08 | 8.08 | ± | 6.51 |

Lipid mediators were extracted, identified and quantified in plasma from IR Late and IR early patients using Lipid Mediator profiling. Results are mean ± SEM. n = 9 IR Late and 8 IR early patients. - = Below limits of the assay
